# Knowledge of Neonatal Danger Signs Among Mothers of Neonates in and Around Chennai: A Cross-Sectional Study

**DOI:** 10.7759/cureus.83798

**Published:** 2025-05-09

**Authors:** Swaminathan Ramasubramanian, Angel Saravana Priya Annamalai Pandian, Ravi Sundararaj, Vidhyashree Murugan Dhanapal, Arun Murugan, Tharika Rajendran, Vinitha Baskaran, Snehanjali Balamurugan, Subind Srinivas Elango, Raashidha Subramanian

**Affiliations:** 1 Epidemiology and Public Health, Government Medical College, Omandurar Government Estate, Chennai, IND; 2 Epidemiology and Public Health, Government Kilpauk Medical College, Chennai, IND; 3 Epidemiology and Public Health, Government Vellore Medical College, Vellore, IND; 4 Pediatrics, Medway Hospitals, Mogappair, Chennai, IND

**Keywords:** chennai, maternal knowledge, neonatal danger signs, neonatal mortality, public health, socio-economic factors

## Abstract

Background

Neonatal mortality remains a significant public health issue globally, particularly in low- and middle-income countries such as India. Early identification of neonatal danger signs (NDSs) is vital for reducing mortality rates, yet many mothers lack the knowledge required to recognize these warning signs. This study aims to assess the knowledge of NDSs among mothers of neonates in Chennai and nearby areas.

Objective

To estimate the level of knowledge of NDSs among mothers in and around Chennai, and to analyze the association between maternal age, socio-economic factors, healthcare availability, and employment status with their knowledge of NDSs.

Methods

A cross-sectional study was conducted between August and December 2022, across three healthcare facilities in Chennai. Data were collected from 430 postnatal mothers using a standardized, interviewer-administered questionnaire. Participants were selected through convenience sampling. The data were analyzed using descriptive statistics, chi-square tests, logistic regression, and non-parametric methods, with analysis performed in Python version 3.9 (Python Software Foundation, Wilmington, DE, USA).

Results

Among the 430 mothers, the average age was 26 years, with 321 (74.65%) being non-working. High awareness was observed for fever (91.86%, n = 395), diarrhea (74.88%, n = 322), and vomiting (69.53%, n = 299), while signs such as hypothermia (26.51%, n = 114) and lethargy (38.60%, n = 166) were poorly recognized. Family income was significantly associated with higher maternal knowledge of NDSs. However, maternal age, education level, employment status, antenatal visits, and spouse accompaniment did not demonstrate significant associations in the adjusted analysis.

Conclusion

Socio-economic status and healthcare access were significantly linked to maternal knowledge of NDSs. Despite high recognition of some danger signs, critical signs like hypothermia were poorly recognized. Tailored public health interventions focusing on economically disadvantaged groups and comprehensive health education could bridge these knowledge gaps, potentially reducing neonatal mortality in Chennai.

## Introduction

Neonatal mortality remains a critical public health challenge globally, with profound implications for societal development and family well-being. Despite significant advancements in healthcare, neonatal deaths continue to account for a substantial proportion of under-five mortality, particularly in low- and middle-income countries like India [1,2]. Timely identification and response to neonatal danger signs (NDSs) are pivotal in reducing neonatal mortality rates. Delays in seeking medical care, often attributable to inadequate recognition of these danger signs, are among the leading contributors to increased infant mortality [3,4]. In India, the Infant Mortality Rate (IMR) stood at 20.35 per 1,000 live births in 2020, reflecting persistent disparities in child health outcomes [5]. The Millennium Development Goal 4 (MDG 4) emphasized reducing child mortality by two-thirds, a target that underscores the necessity of early healthcare interventions at the neonatal stage [6]. Achieving this goal requires not only the availability of healthcare services but also the empowerment of mothers with the knowledge to recognize and act upon NDSs promptly.

NDSs encompass a range of clinical indicators that suggest severe illness in newborns, necessitating immediate medical attention. These include difficulty in feeding, convulsions, rapid breathing (defined as 60 breaths per minute or more), severe chest indrawing, fever (temperature above 37.5°C), hypothermia (temperature below 35.4°C), yellowing of the soles and sclera, reduced or absent movement, and signs of local infections, among others [7,8]. Awareness of these signs among mothers is crucial, as early detection and timely medical intervention can significantly improve neonatal outcomes and reduce mortality rates. Despite the critical importance of maternal knowledge in recognizing NDSs, there is limited comprehensive data on the extent of this awareness among mothers, particularly in urban and semi-urban settings like Chennai. Previous studies have highlighted varying levels of awareness, influenced by factors such as education, socioeconomic status, and access to healthcare services [4,7,9-11]. However, there remains a gap in understanding how these factors interplay specifically within the context of Chennai, a metropolitan area with diverse populations and healthcare infrastructures. Socio-economic disparities often exacerbate the challenges in neonatal care. Mothers from lower socioeconomic backgrounds may have limited access to information and healthcare resources, hindering their ability to recognize and respond to danger signs effectively. Additionally, the availability and accessibility of healthcare facilities play a significant role in determining the promptness of medical care sought by mothers when faced with neonatal health issues. Understanding these dynamics is essential for designing targeted interventions that address the specific needs of different demographic groups.

Another critical aspect is the role of maternal employment in shaping health-seeking behaviors. Working mothers may face time constraints and logistical challenges that impact their ability to seek timely medical care for their neonates. Conversely, non-working mothers might have more flexibility but could lack the necessary support systems or information to recognize danger signs. Exploring these nuances can provide valuable insights into tailoring health education and support mechanisms to different maternal profiles. Addressing these knowledge gaps is imperative for developing effective public health strategies aimed at reducing neonatal mortality. By assessing the current level of awareness among mothers in and around Chennai, this study seeks to inform policymakers and healthcare providers about the areas needing attention and improvement. The primary objective of this study is to estimate the prevalence of knowledge regarding NDSs among mothers residing in and around Chennai. Additionally, the study aims to explore several secondary objectives: to assess the association between maternal age and knowledge of NDSs; to examine how socio-economic factors influence this knowledge; to evaluate the impact of healthcare availability on maternal awareness; and to compare the level of knowledge between working and non-working mothers.

## Materials and methods

Study design

This study was designed as a cross-sectional survey to estimate the level of knowledge regarding NDSs among postnatal mothers.

Study period and center

The study was conducted over five months, from August to December 2022, across three government-affiliated urban healthcare facilities in Chennai, Tamil Nadu. These included the Government Medical College and Hospital, Omandurar Estate - a tertiary care referral center; the Institute of Social Obstetrics and Government Kasturba Gandhi Hospital - a specialized facility for maternal health; and the Urban Primary Health Centre in Pudupet, which caters to lower-income urban populations. The selection of these centers ensured representation from varied socio-economic and healthcare access backgrounds.

Study population

The study population comprised postnatal mothers who had delivered within the past 42 days and were visiting the selected healthcare facilities for postnatal care, immunization, or neonatal follow-up during the data collection period. Convenience sampling was employed due to the logistical constraints and the setting-based recruitment of eligible participants during routine clinic hours.

Inclusion criteria

Mothers were included in the study if they were at least 18 years of age, had delivered a live-born infant within the past 42 days, and were residents of Chennai or the surrounding peri-urban regions within a 50-kilometer radius. Only those who provided informed written consent and were the primary caregivers of the neonate during the neonatal period were enrolled.

Exclusion criteria

Exclusion criteria comprised mothers whose neonates had been hospitalized for more than 14 consecutive days in a neonatal intensive care unit (to avoid bias arising from increased healthcare exposure), those with acute or chronic cognitive impairments or psychiatric conditions that could compromise comprehension, and those who declined participation.

Sample size estimation

Using a prior study done by Zhou et al. [3], which reported a 52% prevalence of adequate knowledge of NDSs, the sample size was calculated using the formula: $n = \frac{4pq}{L^2}$ where, p = 0.52, q = 1 - p = 0.48 and L = 0.05 (allowable error, 5%). Substituting these values gives:

$n = (4 &times; 0.52 &times; 0.48)/0.0025=399.36$

After accounting for design effect, non-response, and potential dropouts, the final sample size was rounded to 430 mothers.

Data collection procedure

Data were collected using a structured, interviewer-administered questionnaire. The questionnaire was initially prepared in English and then translated into Tamil to ensure that all participants could fully understand and accurately respond to the questions. The translation was carefully done to maintain the content validity of the instrument, and it was pretested to ensure clarity and cultural appropriateness. The questionnaire was adapted and modified from previous studies on similar topics, with modifications to suit the local context and the specific objectives of this study [3,4,11-13]. The questionnaire covered various aspects, including demographic information, socioeconomic status, healthcare access, and specific knowledge of NDSs. Mothers were considered to have adequate knowledge if they could identify at least three NDSs. This threshold was based on existing literature and expert recommendations, ensuring that the criteria for adequate knowledge were both evidence-based and contextually relevant. After completing the interviews, the data were carefully transcribed into a Google Form.

Ethical considerations

The study protocol was reviewed and approved by the Institutional Ethics Committee (approval no. 85/IEC/GOMC/2022), and written informed consent was obtained from all participants. Confidentiality and anonymity were ensured throughout data handling and analysis.

Statistical analysis

Descriptive statistics were employed to profile the sample. Categorical variables, including maternal education, occupation, family income, and awareness of specific NDSs, were summarized using frequencies and percentages. Bivariate analysis was performed to explore associations between variables. The chi-square test was used to examine relationships between categorical independent variables, such as maternal education and family income, and the dichotomized dependent variable representing knowledge levels (categorized as 'Low' and 'High'). For continuous variables, either the Mann-Whitney U test or the T-test was applied, depending on the data distribution, to compare knowledge levels across groups. Knowledge scores were classified into 'Low' (score < 8) and 'High' (score ≥ 8), based on the sample's mean score. Multivariate logistic regression analysis was conducted to identify independent predictors of high knowledge levels regarding NDSs. Both Crude Odds Ratios (CORs) and Adjusted Odds Ratios (AORs) were calculated. Variables that showed significant associations (p < 0.05) in the bivariate analysis, such as family income, Kuppuswamy Scale Score [14], maternal education, and other socio-demographic factors, were included in the multivariate model. The model’s goodness-of-fit was evaluated using the Hosmer-Lemeshow test. Additionally, the Number Needed to Treat (NNT) was computed for significant predictors. All statistical analyses were conducted using Python version 3.9 (Python Software Foundation, Wilmington, DE, USA), utilizing libraries such as numpy, scipy, pandas, matplotlib, and seaborn.

## Results

This study encompassed 430 mothers, whose demographic and socio-economic characteristics are detailed in Table 1. The average age of the mothers was approximately 26 years (mean = 25.98, SD = 4.13), and the mean birth weight of their newborns was 2.72 kg (SD = 0.41). Regarding maternal education, 20 (4.65%) of mothers were illiterate, while 35 (8.14%) were literate without formal schooling. A further six (1.40%) had completed primary school, and 107 (24.88%) had attained education up to middle school. High school certification was held by 73 (16.98%) of the mothers, whereas 47 (10.93%) had studied up to the intermediate level. Additionally, 123 (28.60%) were graduates, and 19 (4.41%) had completed professional degrees. The majority of mothers were non-working (74.65%, n = 321), while 109 (25.35%) were employed. The median number of antenatal visits was 9, with an interquartile range of 6-10. The median family income was between INR 10,357 and INR 15,535 per month, and the mean Kuppuswamy Scale Score [14] was 13.94 (SD = 6.04), indicating a distribution across different socio-economic classes. The Kuppuswamy Scale [14] categorization revealed that 160 (37.21%) of the participants belonged to the Upper Lower Class, 115 (26.74%) to the Upper Middle Class, 116 (26.98%) to the Lower Middle Class, and 39 (9.07%) to the Upper Class, as detailed in Table 1. Antenatal visits had a median of 9 (IQR: 6-10). Family income categories showed that 30 (6.98%) of families earned above INR 41,430 per month, while 36 (8.37%) earned below INR 2,091 per month, with the remaining families distributed across intermediate income brackets (Table 1).

**Table 1:** Demographic and Socio-Economic Characteristics of Mothers INR, Indian Rupees

Variable	Count	Frequency (%)
Mother's Education Level		
Illiterate	20	4.65%
Literate	35	8.14%
Primary School	6	1.40%
Middle School	107	24.88%
High School Certificate	73	16.98%
Intermediate	47	10.93%
Graduate	123	28.60%
Professional	19	4.41%
Mother's Occupation		
Working	109	25.35%
Non-working	321	74.65%
Kuppuswamy Scale [14]		
Upper Class	39	9.07%
Upper Middle Class	115	26.74%
Lower Middle Class	116	26.98%
Upper Lower Class	160	37.21%
Lower Class	0	0.00%
Family Income Categories (INR)		
>41,430	30	6.98%
20,715-41,429	107	24.88%
15,536-20,714	54	12.56%
10,357-15,535	106	24.65%
6,214-10,356	90	20.93%
2,092-6,213	7	1.63%
<2,091	36	8.37%

**Figure 1:**
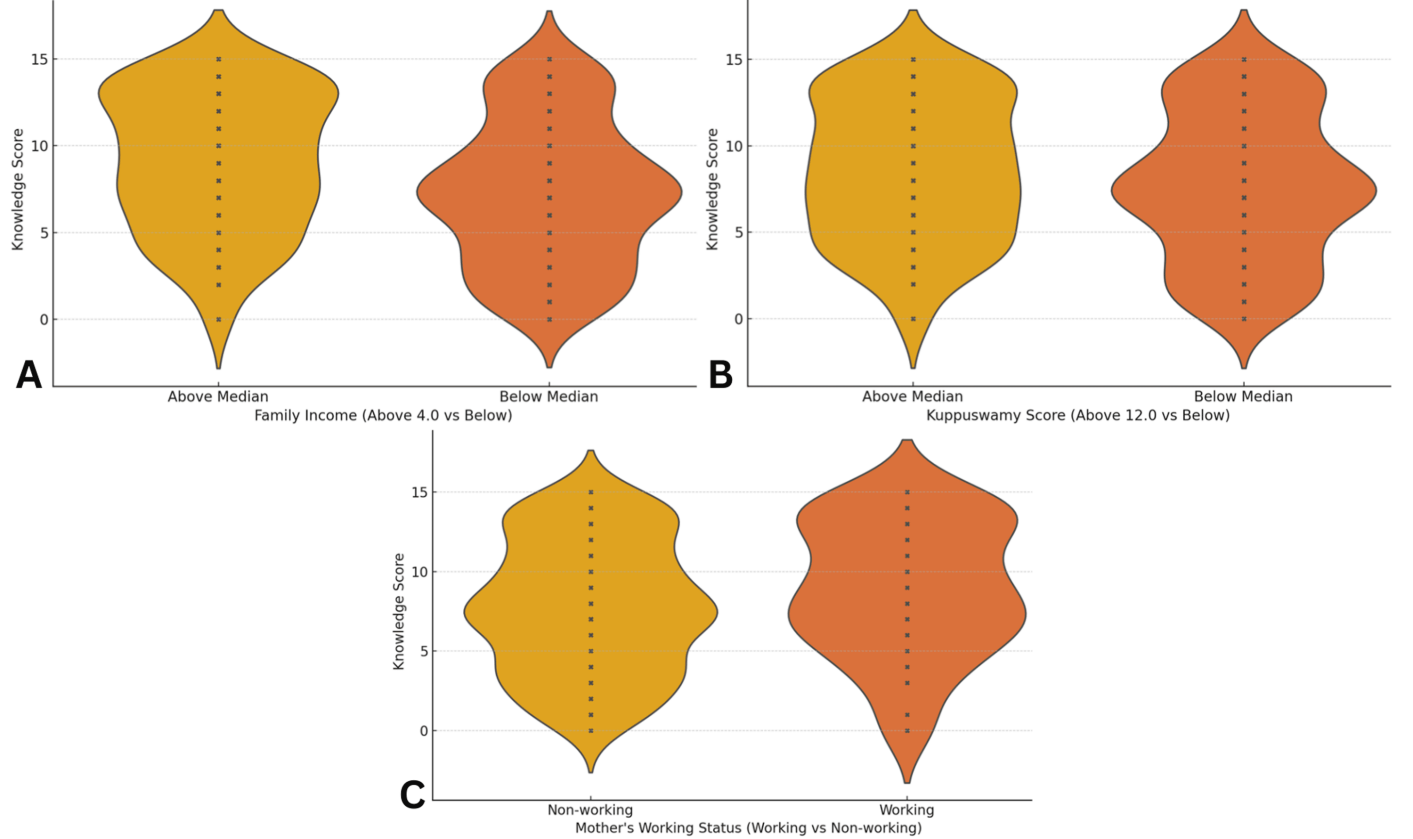
Distribution of Knowledge Score (A) Family Income; (B) Kuppuswamy Score [14]; (C) Mother's Working Status

Awareness of NDSs among the mothers is presented in Table 2. Notably, fever was the most recognized NDS, with 395 (91.86%) of mothers aware of it, followed by diarrhea (74.88%, n = 322) and vomiting (69.53%, n = 299). In contrast, awareness was lower for hypothermia (26.51%, n = 114), lethargy (38.60%, n = 166), cyanosis (38.37%, n = 165), abdominal distention (40.23%, n = 173), excessive weight loss (40.47%, n = 174), and signs of local infection (36.98%, n = 159). Other danger signs, such as refusal to feed (55.12%, n = 237), increased breathing rate (62.33%, n = 268), increased breathing effort (60.70%, n = 261), convulsions (59.77%, n = 257), bleeding (51.86%, n = 223), and yellow soles and/or palms (56.05%, n = 241), showed moderate levels of awareness among the participants. These findings are visually represented in Figure 1.

**Table 2:** Awareness of Neonatal Danger Signs Among Mothers

Neonatal Danger Sign	Number of Mothers Aware	Number of Mothers Unaware	Total Mothers	Percentage Aware (%)
Lethargy	166	264	430	38.60%
Fever	395	35	430	91.86%
Hypothermia	114	316	430	26.51%
Refusal to Feed	237	193	430	55.12%
Increased Breathing Rate	268	162	430	62.33%
Increased Breathing Effort	261	169	430	60.70%
Cyanosis	165	265	430	38.37%
Convulsions	257	173	430	59.77%
Abdominal Distention	173	257	430	40.23%
Bleeding	223	207	430	51.86%
Yellow Soles and/or Palms	241	189	430	56.05%
Excessive Weight Loss	174	256	430	40.47%
Vomiting	299	131	430	69.53%
Diarrhea	322	108	430	74.88%
Signs of Local Infection	159	271	430	36.98%

The normality of the data was assessed using Shapiro-Wilk tests, which revealed that continuous variables, such as Age, Knowledge Score, and Kuppuswamy Scale Score [14], did not follow a normal distribution (p < 0.05 for each). Due to these non-normal distributions, non-parametric statistical methods were employed throughout the analysis to ensure the validity of the results. To examine the associations between socio-demographic and healthcare factors with the knowledge levels of NDSs, chi-square tests of independence were primarily utilized. In cases where the expected frequencies in contingency tables were below five, Fisher's exact test was implemented as an alternative to maintain the accuracy of the statistical inferences. Logistic regression analyses were conducted to calculate CORs and AORs, allowing for the assessment of both unadjusted and adjusted relationships between the independent variables and the categorized knowledge scores.

The relationship between maternal knowledge of NDSs and various socio-demographic and healthcare factors was analyzed based on a binary knowledge score classification (‘Low’ <8 and ‘High’ ≥8). The consolidated results are presented in Table 3. No statistically significant association was observed between maternal age and knowledge scores. While mothers aged 26-30 years (n = 136) and those above 30 (n = 67) had relatively higher proportions of high knowledge - 79 (58.09%) and 38 (56.72%), respectively - the differences across age groups were not significant (all p > 0.1). Educational level yielded some significant findings. Mothers with basic literacy (n = 35) showed a markedly higher proportion of high knowledge scores (n = 28; 80.00%, p = 0.0017) compared to illiterate mothers (n = 20; 9 with high knowledge, 45.00%). Similarly, intermediate-level education (n = 47; 35 with high knowledge, 74.47%, p = 0.0034) and graduate-level education (n = 123; 55 with high knowledge, 44.72%, p = 0.0324) were associated with significantly better knowledge. Family income demonstrated the most striking associations. Among mothers in the lowest income bracket (<INR 2,091; n = 36), only five (13.89%) had high knowledge (p = 0.00000183). Conversely, in the INR 15,536-20,714 bracket (n = 54), 45 mothers (83.33%) had high knowledge (p = 0.0000044), indicating a strong association between income level and awareness. Socio-economic status also showed significant correlations. Mothers in the Lower Middle Class (n = 116) had 74 (63.79%) high knowledge scores (p = 0.0107), while those in the Upper Lower Class (n = 160) had significantly lower awareness, with only 70 (43.75%) scoring high (p = 0.0033). Although not statistically significant (p = 0.0978), a higher proportion of working mothers (n = 109; 66 with high knowledge, 60.55%) had better knowledge than non-working mothers (n = 321; 163 with high knowledge, 50.78%). Healthcare access indicators, including a number of antenatal visits and spouse accompaniment, showed no significant association with knowledge levels (p > 0.28). Among sources of information, Village Health Nurses were the most frequently reported (n = 189; 44%), followed by Doctors (n = 172; 40%). Staff Nurses (n = 52; 12%) and Social Media (n = 17; 4%) played comparatively minor roles.

**Table 3:** Association Between Maternal and Household Factors and Knowledge Levels ^ denotes Fisher's Exact Test value; * denotes p < 0.05 INR, Indian Rupees

Variable	Category	n	Low Knowledge (%)	High Knowledge (%)	χ²-value	p-value
Maternal Age (Years)	<20	18	66.67%	33.33%	2.19	0.1389
	20-25	208	49.52%	50.48%	0.95	0.3287
	26-30	136	41.91%	58.09%	1.67	0.1959
	>30	67	43.28%	56.72%	0.25	0.6142
Education Level	Illiterate	20	55.00%	45.00%	0.28	0.5973
	Literate	35	20.00%	80.00%	9.84	0.0017*
	Primary School	6	66.67%	33.33%	0.33^	0.5667
	Middle School	107	51.40%	48.60%	1.00	0.3162
	High School Certificate	73	47.95%	52.05%	0.01	0.9227
	Intermediate	47	25.53%	74.47%	8.61	0.0034*
	Graduate	123	55.28%	44.72%	4.57	0.0324*
	Professional	19	47.37%	52.63%	0.00^	1.0000
Family Income (INR/Month)	<2,091	36	86.11%	13.89%	22.61	0.00000183*
	2,092-6,213	7	42.86%	57.14%	0.00^	1.0000
	6,214-10,356	90	52.22%	47.78%	1.11	0.2925
	10,357-15,535	106	47.17%	52.83%	0.00	1.0000
	15,536-20,714	54	16.67%	83.33%	21.26	0.0000044*
	20,715-41,429	107	46.73%	53.27%	0.00	1.0000
	>41,430	30	36.67%	63.33%	0.92	0.3384
Kuppuswamy Scale [14]	Upper Class	39	43.59%	56.41%	0.06	0.8059
	Upper Middle	115	45.22%	54.78%	0.08	0.7839
	Lower Middle	116	36.21%	63.79%	6.52	0.0107*
	Upper Lower	160	56.25%	43.75%	8.66	0.0033*
Occupation	Non-working	321	49.22%	50.78%	2.74	0.0978
	Working	109	39.45%	60.55%	2.74	0.0978
Healthcare Access	<4 Antenatal Visits	14	64.29%	35.71%	1.13^	0.2868
	≥4 Antenatal Visits	416	46.15%	53.85%	1.13^	0.2868
	Spouse Not Accompanied	96	47.92%	52.08%	0.02	0.8846
	Spouse Accompanied	334	46.41%	53.59%	0.02	0.8846

The multivariable analysis presented in Tables 4-5 highlights significant associations between maternal knowledge of NDSs and two key factors: family income and socio-economic status. Higher family income was independently associated with greater knowledge (AOR = 1.61; 95% CI: 1.32-2.44; p = 0.0249), with a Number Needed to Treat (NNT) of 1.64 - indicating that for every two mothers from higher-income families, one is likely to have a high knowledge score. Similarly, a higher Kuppuswamy Score [14], reflecting better socio-economic status, was negatively associated with increased knowledge (AOR = 0.77; 95% CI: 0.50-0.98; p = 0.0320; NNT = 4.33). While crude analysis suggested that higher paternal education was inversely associated with maternal knowledge (COR = 0.59; p = 0.0097), this association lost significance after adjustment (AOR = 1.095; p = 0.6315). Other variables, including maternal age, maternal education beyond primary level, employment status, number of antenatal visits, and spouse accompaniment, showed no significant associations in the adjusted model.

**Table 4:** Bivariate Analysis COR, Crude Odds Ratio; CI, Confidence Interval; NNT, Number Needed to Treat

Factor	COR	CI Lower (COR)	CI Upper (COR)	Wald χ²	p-value (COR)	NNT (COR)
Age Category	1.397	0.954	2.045	2.95	0.0856	2.52
Mother's Education	1.025	0.700	1.501	0.02	0.8987	39.86
Father's Education	0.590	0.395	0.880	6.68	0.0097	2.44
Family Income	0.477	0.323	0.704	13.85	0.0002	1.91
Kuppuswamy Score [14]	0.681	0.464	0.999	3.87	0.0491	3.13
Mother's Working Status	1.488	0.956	2.315	3.10	0.0782	2.05
Antenatal Visits	0.476	0.157	1.445	1.72	0.1902	1.91
Spouse Accompaniment	0.941	0.597	1.483	0.07	0.7939	17.01

**Table 5:** Multivariate Logistic Regression AOR, Adjusted Odds Ratio; CI, Confidence Interval; NNT, Number Needed to Treat

Factor	AOR	CI Lower (AOR)	CI Upper (AOR)	Wald χ²	p-value (AOR)	NNT (AOR)
Father's Education	1.095	0.995	1.583	0.23	0.6315	10.58
Family Income	1.610	1.319	2.440	5.03	0.0249	1.64
Kuppuswamy Score [14]	0.769	0.497	0.978	4.60	0.0320	4.33

**Figure 2:**
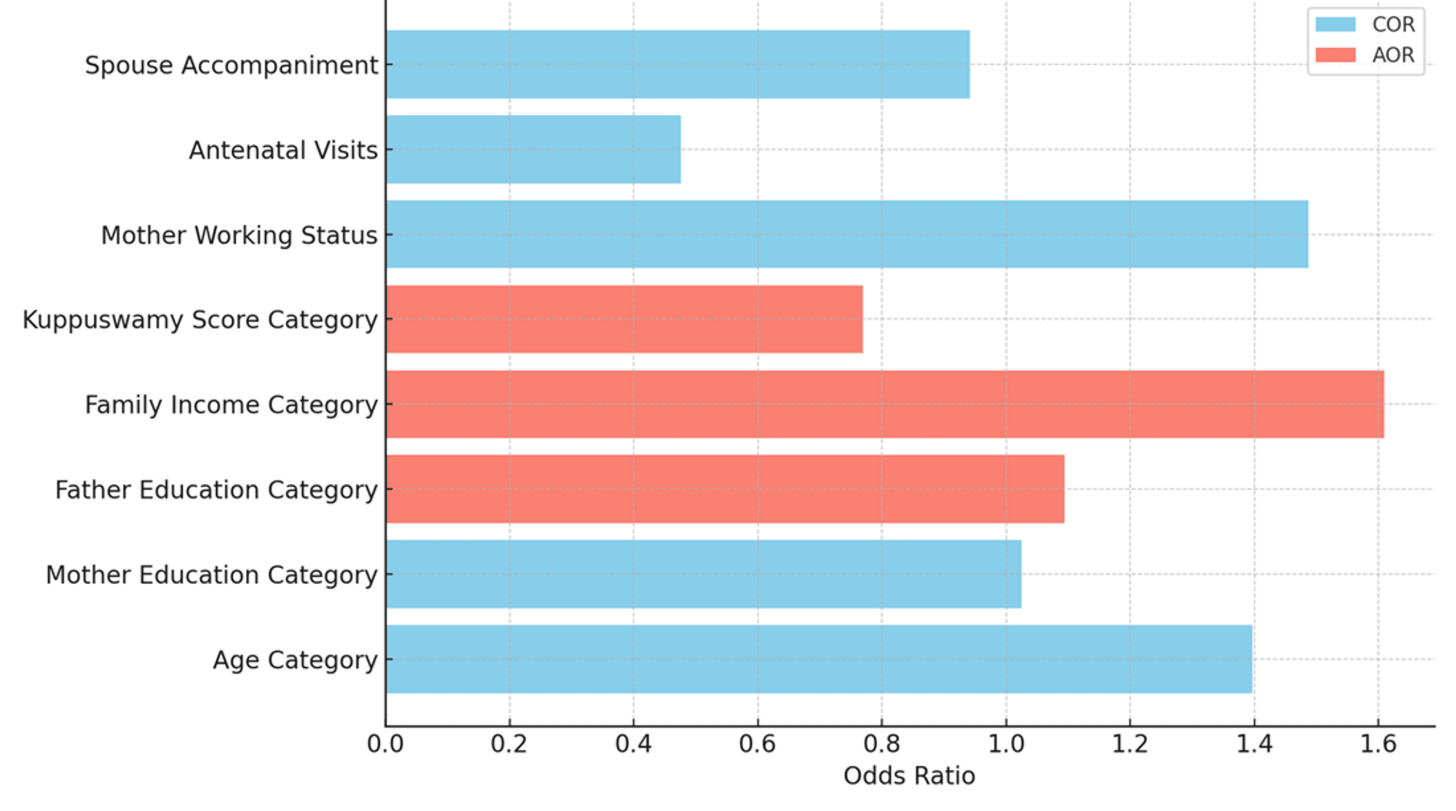
Comparison of COR and AOR for Each Factor COR, Crude Odds Ratio; AOR, Adjusted Odds Ratio

The Hosmer-Lemeshow Goodness-of-Fit Test was performed to assess the fit and generalizability of the logistic regression model. The test resulted in a Hosmer-Lemeshow statistic of 2.23 (df = 8), with a p-value of 0.093. Given that the p-value is greater than 0.05, there is no significant discrepancy between the observed and expected values, suggesting that the model fits the data adequately and is likely applicable to similar populations. Figure 2 compares the COR and AOR for each variable, offering a visual representation of the strength and direction of these associations.

## Discussion

This study assessed the awareness of NDSs among 430 mothers, focusing on demographic and socio-economic determinants influencing their knowledge levels. The average age of the mothers was approximately 26 years, and the majority (74.65%, n = 321) were non-working. Educational attainment varied, with 24.88% holding higher secondary education and 16.98% possessing graduate or postgraduate degrees. The socio-economic status, as measured by the Kuppuswamy Scale [14], indicated that 37.21% (n = 160) of the participants belonged to the Upper Lower Class. Awareness of NDSs was notably high for fever (91.86%, n = 395), diarrhea (74.88%, n = 322), and vomiting (69.53%, n = 299), whereas awareness was significantly lower for signs such as hypothermia (26.51%, n = 114) and lethargy (38.60%, n = 166). Statistical analysis revealed that higher family income was significantly associated with higher knowledge scores. Proximity to private healthcare facilities was linked to greater awareness of NDSs. However, factors such as maternal age, overall education level, employment status, antenatal visits, and spouse accompaniment during visits did not show significant associations with knowledge levels after adjusting for other variables.

The findings of this study align with several previous studies that highlight the critical role of socio-economic factors in maternal knowledge of NDSs. For instance, the study by Bayih et al. [9] in Ethiopia found that maternal education and antenatal care visits were significant predictors of good knowledge of NDSs - similar to our findings, where higher family income was associated with better knowledge scores. However, unlike some studies that identified maternal education as a strong independent predictor, our study found that while overall maternal education did not significantly associate with knowledge levels, specific education levels (primary, intermediate, and graduate) did show significant associations when examined in detail [15,16]. Contrastingly, studies such as those conducted by Abu-Shaheen et al. [17] in Saudi Arabia and Bekele et al. [11] in Ethiopia reported varying levels of knowledge about NDSs, often influenced by factors like postnatal care and institutional delivery. Our study reinforces these findings by demonstrating that socio-economic status, rather than education alone, plays a pivotal role in maternal awareness. This suggests that broader socio-economic interventions may be necessary to enhance knowledge about NDSs beyond educational initiatives. The high recognition of fever as an NDS in our study (91.86%) is consistent with other studies, such as those by Degefa et al. [10] and Bekele et al. [11], where a majority of participants commonly identified fever. The low awareness of hypothermia and lethargy in our study concurs with some literature, where these signs were not recognized widely, despite possible regional or cultural differences in awareness and education regarding specific NDSs [18,19].

The association between proximity to private healthcare facilities and higher knowledge levels echoes findings from previous research, which suggests that access to quality healthcare services enhances maternal knowledge. This aligns with the study by Abu-Shaheen et al. [17], which emphasized the importance of healthcare access in improving awareness of NDSs. Conversely, our study found no significant association between antenatal visits and knowledge levels after adjustment, which differs from studies like Bayih et al. [9], suggesting that the quality and content of antenatal education might vary significantly across different settings.

The significant association between higher family income and better knowledge of NDSs underscores the need for socio-economic interventions aimed at reducing disparities in maternal health education. Enhancing socio-economic status could indirectly improve maternal knowledge, leading to better neonatal health outcomes. Additionally, the positive correlation between proximity to private healthcare facilities and awareness of NDSs highlights the importance of accessible, quality healthcare services in disseminating critical health information. The low awareness of several NDSs - particularly hypothermia and lethargy - indicates gaps in current maternal education programs. Healthcare providers should prioritize comprehensive education that covers a broader range of NDSs, ensuring mothers are well-equipped to recognize and respond to various neonatal health issues. Tailoring educational interventions to target lower socio-economic groups and those with limited access to private healthcare could bridge the knowledge gap identified in this study.

While this study provides valuable insights into the factors influencing maternal knowledge of NDSs, it is not without limitations. The cross-sectional design limits the ability to establish causality between socio-economic factors and knowledge levels. The reliance on self-reported data may introduce response biases, such as social desirability bias, potentially affecting the accuracy of the reported knowledge levels. The study was conducted in a specific geographical area and over a span of five months, which may limit the generalizability of the findings to other regions with different socio-economic and healthcare contexts. Future research should consider longitudinal studies to better understand the causal relationships between socio-economic factors and maternal knowledge of NDSs. Additionally, qualitative studies could explore the underlying reasons for low awareness of specific danger signs, providing deeper insights into cultural, educational, and systemic barriers. Expanding the study to include diverse regions and healthcare settings would enhance the generalizability of the findings and inform more targeted interventions.

## Conclusions

This study highlights significant gaps in maternal knowledge of NDSs, emphasizing the crucial role of socio-economic status and healthcare accessibility in shaping awareness. Despite high recognition of some danger signs, like fever, awareness of critical indicators such as hypothermia and lethargy was notably low. The findings indicate that improved socio-economic conditions and access to quality healthcare facilities are associated with higher maternal knowledge. Educational interventions should therefore focus on economically disadvantaged mothers and emphasize comprehensive neonatal health education to bridge these gaps. The absence of significant associations between factors like maternal occupation or antenatal visits suggests that mere contact with healthcare systems is insufficient - quality, content, and tailored approaches in health education are key. Addressing these disparities through targeted, context-specific public health initiatives is essential to reducing neonatal mortality and improving overall neonatal health outcomes.
